# The Impact of Probe Angle and Swivel Length on Contact Point Identification in Coordinate Measuring Machine Measurements: A Case Study

**DOI:** 10.3390/s25072008

**Published:** 2025-03-23

**Authors:** Tomasz Mazur, Tomasz Szymanski, Waldemar Samociuk, Miroslaw Rucki, Tomasz Ryba

**Affiliations:** 1 Faculty of Mechanical Engineering, Casimir Pulaski Radom University, ul. Stasieckiego 54, 26-600 Radom, Poland; 2 Mitutoyo Polska Sp. z o.o., ul. Graniczna 8A, 54-610 Wrocław, Poland; 3 Faculty of Production Engineering, University of Life Sciences in Lublin, Gleboka 28, 20-612 Lublin, Poland; 4 Institute of Mechanical Science, Vilnius Gediminas Technical University, Sauletekio al. 11, LT-10223 Vilnius, Lithuania

**Keywords:** coordinate measuring machine, precision metrology, accuracy, probing point, error minimization

## Abstract

This paper presents the results of investigations on the accuracy of contact point identification during coordinate measurement, which is crucial in the context of the Industry 4.0 concept. In particular, the effects of swivel length and probe declination angle during measurement were analyzed. In the experiments, deviations from the expected coordinates (0,0,0) of the contact point were analyzed for different rotational angles of the probing head. It was found that the recommended vertical positioning of the stylus at an angle of *A* = 0° might have introduced some insignificant errors. Increasing angle *A* up to 15° generated additional errors of negligible values in comparison with the measurement accuracy of the CMM. However, an increase in angle *A* up to 90° introduced additional errors as high as 10 μm. This contact point identification error will have a certain effect on the best fitting element and subsequent calculations and on the respective measurement results.

## 1. Introduction

Industry 4.0 (I4.0) can be understood as a fusion of a range of concepts and technologies with the integration of digital and physical domains, including biological functions [1]. The development strategy pointed out by I4.0 aims to strengthen the competitiveness of the manufacturing sector [2]. To a large extent, it is connected with the potential of I4.0 for creating smart products [3] and the implementation of smart factories [4,5,6]. Obviously, these require measurement tools as key performance indicators (KPIs) to carry out the monitoring, comparison, and supervision of processes [7].

The transformation of industrial enterprises within the framework of the I4.0 concept towards the introduction of smart manufacturing systems and smart factories is based on the connectivity, virtualization, and utilization of the collected data [8], including the digitization [9], recording, and analysis of large sets of measurement and diagnostic data, or the use of artificial intelligence [10]. Thus, modern machines and industrial lines are equipped with various sensor-based systems to register the parameters of processes, supervise quality, and optimize processes [11]. According to the objectives of Industry 4.0, efforts are being undertaken to improve process automation and to enable the application of predictive maintenance [12,13]. In this context, metrology should be transformed in order to meet the requirements of I4.0 [14] and to enable a transition to the smart factory concept [15]. This extensive review of relevant papers [16] demonstrates significant changes in the role of metrology on the manufacturing shop floor, with increasing applications of in-process monitoring and in-line measurements.

In recent decades, dimensional and form metrology related to I4.0 has become increasingly dependent on coordinate measurement techniques [17]. Perhaps the highest requirements for the accuracy and repeatability of measuring systems, including coordinate measuring machines (CMMs), can be found in the aerospace and automotive industries [18]. The combined calibration techniques applied in order to improve milling precision may use an external CMM-based measurement system with a relevant compensation method [19]. CMMs have been used in the retrofitting of CNC equipment with the aim of achieving Industry 4.0-level connectivity and sustainability [20]. Measurement with CMMs can be involved in the creation of a Cyber–Physical Manufacturing Metrology Model for the integration of digital product metrology and the generation of an inspection plan [21]. The creation of new interface definitions within the framework of I4.0 is focused on improved information exchange between digital programs analyzing the dimensional quality, including the measurement data obtained from CMMs [22].

In this context, the I4.0 concept requires improved accuracy and reliability of CMM measurements. There are many reports on the accuracy analysis of CMMs, including the analysis of different types of axis errors [23], the accuracy of planar point identification [24], spatial geometric errors [25], dynamic errors [26], probe ball tip form errors [27], direction-dependent errors [28], or errors occurring after the transformations of coordinate systems [29]. Error evaluation can be related to the coordinate measurement of curved profiles [30] or freeform surface profiles [31]. There are also reports on accuracy analysis of the roundness deviation measurement, exploring the effect of the stylus tip on measurement results, depending on the roughness of the measured surface [32]. Watanabe and co-authors proposed a novel, accurate measurement method for industrial parts with a curvature radius smaller than a few millimeters [33]. Urban with his team performed an analysis of the optimization of CMM measurement plans in order to reduce the measurement error, considering the environmental impact and economic benefits [34]. Also, the effect of the part coordinate system on measurement errors has been analyzed [35].

Usually, the measurement of points placed on a plane with probes of different rotation angles, keeping the perpendicularity of the approach direction to the tested plane, provides repeatable results, consistent with expectations. However, when the points are placed on a cylindrical surface, the same measurement conditions provide very different results in practical applications. At the same time, no data are available on the effect of probe angle and swivel length on measurement results. Thus, it is necessary to perform a quantitative analysis of the effect on the determined coordinates of relevant points when the recommended approach direction is kept along the curvature radius.

In the current research, the objective was to investigate the impact of the swivel length of the probe and the stylus’s rotation angle on the results of tactile CMM measurement. The most attention was paid to the direction of contact between the probe’s ball tip and the measured surface, especially when these were different from those recommended by the CMM’s specifications. Sometimes, it is impossible to follow the recommendations strictly, and the measurement must be performed with the stylus declined by several degrees or even more. This paper presents an attempt to assess the measurement accuracy in these cases.

## 2. Materials and Methods

In order to determine the effect of the probe angle and swivel length on contact point identification, the following experimental conditions were maintained:

- All repetitions were performed at the same point on the shaft surface;
- All repetitions were conducted from one CMM start, without resetting it;
- After the intial start, calibration was performed once for all the repetitions;
- All measurements were carried out in the same Part Coordinate System, defined at the beginning and closely connected with the measurement conditions;
- The fixation and orientation of the measured object were not changed;
- The tested probing point was placed in a position usually applied in measurements due to its convenience.

In the abovementioned framework, the case study was able to minimize the influence of other factors and maximize the repeatability conditions.

### 2.1. Measured Object

In the research, a typical cylindrical element was used. The shaft was made out of tool steel NC11LV delivered by Bohler Uddeholm Polska Sp. z o.o. (Łomianki, Poland), equivalent to 1.2379/X153CrMoV12, and received heat treatment up to HRC 60. The choice of this element was determined by a complimentary research program, where novel ceramic cutting inserts were tested. The shafts obtained from the cutting tests exhibited high accuracy both in dimensions and in shape. The cutting insert was round, with a diameter of 12 mm, a cutting speed of *V_c_* = 300 m/min, a cut material thickness of *a_p_* = 0.2 mm, and a feed rate of 0.15 mm per rotation. During the turning process on the Mazak Quick Turn 100 machine tool (Yamazaki Mazak Corp., Takeda, Japan), the shaft was fixed in a three-jaw universal chuck and supported with a tail stock. No cooling liquid was used.

**Figure 1:**
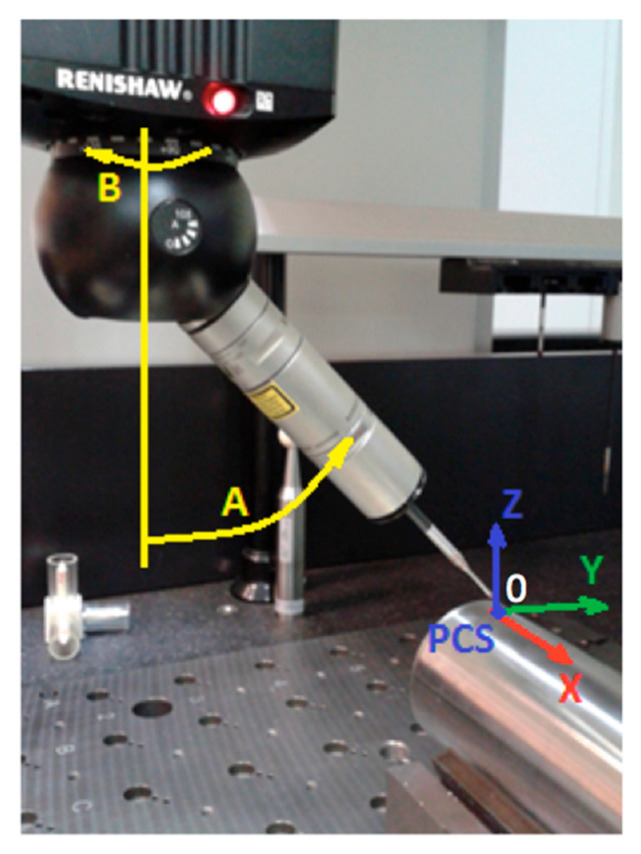
The measured object (shaft) in the CMM’s measuring space. *A* and *B* denote the rotation angles of the probing head. PCS is the Part Coordinate System described in Section 2.3.1.

The diameter of the shaft was 55.85 mm, and the surface roughness was *Ra* = 0.4 μm. The element was placed in the prism on the CMM table, positioning its rotational axis along the OX axis of the CMM’s coordinate system, as shown in Figure 1.

Due to the weight of the measured shaft, it did not require any additional special fixing or orienting elements. During the repetitions, it remained unmoved in exactly the same position.

### 2.2. Coordinate Measuring Machine

**Figure 2:**
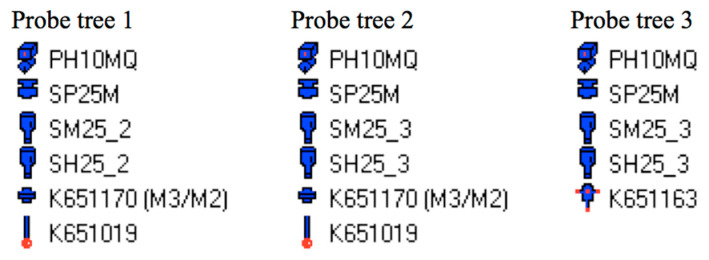
The probe trees used in the experimental research.

The device chosen for the experiments was the Mitutoyo Crysta-Apex C 7106 CMM (Mitutoyo, Kawasaki, Japan) at the Technological Laboratory of the Faculty of Mechanical Engineering, Radom University, Poland. The device has been used since 2012 for experimental research and underwent accuracy verification in August 2024. According to Calibration Certificate No. C24104, the CMM exhibited a maximum permissible error of *MPE_E_*_0_ = ±(1.9 + 4 *L*/1000) μm, depending on the measured length *L* (mm), and a measurement uncertainty of *U* = ±(0.3 + 0.4 *L*/1000). During the experiment, the software Geopak CMM 5.1.0.24903 from the package MCOSMOS-3 ver.5.1 RC3 was used. The measurement was performed with the Renishaw PH10MQ probe head (Renishaw, Wotton-under-Edge, UK). According to the specification [36], it ensures position repeatability (2*σ*) below 0.4 μm. Three probe trees were used, as shown in Figure 2. In all measurements, the probes had the same ball tip diameter of 2 mm, but different swivel lengths.

The configurations of the probe trees were based on the actually available components. The main purpose of the tested probe trees was to compare two probes of similar swivel lengths and a significantly shorter probe in order to determine the effect of the swivel length on the measurement results. The swivel length of the probes from tree #1 was *L_S_*_1_ = 173.35 mm, which was intentionally significantly smaller than the other two, which were *L_S_*_2_ = 257.35 mm and *L_S_*_3_ = 253.85 mm. During the measurement, the rotational angles *A* and *B* of the probe (shown in Figure 1 above) were chosen as follows. First, the stylus was turned around the *z*-axis by subsequent angles of *A* = 0°, 15°, 30°, 45°, 60°, and 90°. The time-consuming repetitions at an angle of 75° were omitted based on the initial measurements that proved no significant deviation from the trend. Secondly, the stylus was turned around the newly determined *x*′-axis by angles of *B* = 90° and 180°. This way, the turned probe was first placed along the shaft axis, corresponding with the OX axis of the CMM, and then perpendicularly to it.

### 2.3. Measurement Strategy

#### 2.3.1. Part Coordinate System

**Figure 3:**
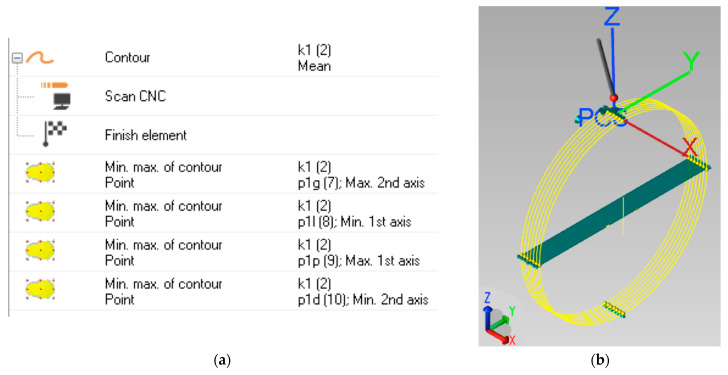
Determination of the Part Coordinate System (PCS): (**a**) determination of quadrants from the scanned circle; (**b**) axes of the PCS.

For the experimental measurements, the Part Coordinate System (PCS) was defined, as shown in Figure 1 above. The stylus was positioned horizontally, with respective angles of *A* = 90° and *B* = 90°, and parallel to the shaft axis. From this position, the profile of the shaft was measured in scanning mode, collecting the respective points on the measured circle in the plane perpendicular to the cylinder axis, with a step size of 0.005 mm. It was denoted as k1(2), as shown in Figure 3a. In this way, the upper, right, left, and lower quadrants of the scanned circle were determined. The notations in Figure 3a are as follows: p1g(7) is the upper quadrant, p1l(8) is the left one, p1p(9) is the right one, and p1d(10) is the lower quadrant.

Next, the scanning procedure was repeated at 6 subsequent intersections, moving the probe forward by 1 mm each time. From the obtained left and right quadrants of each scanned circle, totaling 14 in all, the basic plane OXY was defined. The direction of the OX axis was defined from 7 obtained upper quadrants, and the center of the coordinate system OXYZ was placed in the upper quadrant of the central intersection. The sketch of the PCS determination is presented in Figure 3b. All the quadrants were calculated according to the mean square formula, while the basic elements were calculated according to the minimal zone criterion.

The PCS defined this way became the basis for all performed measurements. It was determined once, after the CMM was launched, and used for all series of measurements, keeping the repeatability conditions recommended by the Joint Committee for Guides in Metrology (JCGM) [37].

#### 2.3.2. Measurement Procedure

**Figure 4:**
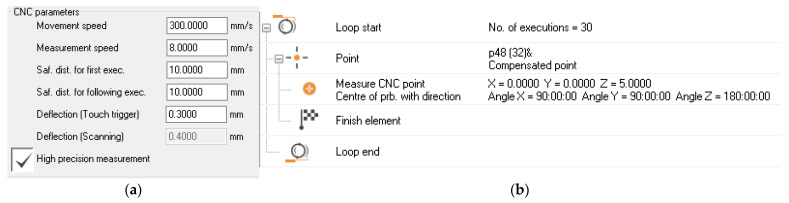
Calibrations and measurements in CNC mode: (**a**) screenshot of the calibration parameters; (**b**) measurement procedure of the point (0,0,0).

Following the probe trees specified in Figure 2 above (Section 2.2), it was necessary to create the corresponding number of probes, calculated as 6 × 2 × 3 = 36. After the CMM was launched, the probes had to be prepared for measurement through calibration in CNC mode. The calibration parameters input to the system can be seen in the screenshot in Figure 4a. Figure 4b illustrates the measurement of the point (0,0,0).

It was decided to perform 30 repetitions of the measurement of the point (0,0,0), with each probe compensated in CNC mode. The measurements were performed with the following settings:

- Movement speed of 520 mm/s;
- Measurement speed of 1.5 mm/s;
- Safety distance of 0.5 mm;
- Starting point of (0,0,5);
- Loop measurement along the PCS axis.

The above mentioned procedure provided a total number of measurements of the point (0,0,0) of 36 × 30 = 1080.

#### 2.3.3. Data Processing

From each set of 30 repetitions, the standard uncertainty was determined and Type A expanded uncertainty was calculated according to the Guide [37]. The uncertainty was compared to the maximum permissible error, *MPE_E_*_0_, in order to make sure it had not been exceeded. The arithmetic mean from the series of repetitions was then compared to the expected true values of the coordinates of the measured point (0,0,0). The difference between the expected value of 0 and the actual indication of the CMM was calculated as the identification error, Δ.

## 3. Results and Discussion

The results obtained from each of the 36 series of measurements were processed by calculating the arithmetic mean value for the 30 repetitions of the point (0,0,0) identification, along with the respective dispersion of the results, as follows:

(1) $$N=\bar{N}\pm \frac{t}{\sqrt{n}}S_{n},$$

where *n* = 30 is the number of repetitions; *t* = 2.676 is the value from the respective Student’s distribution for the confidence level of 99.5% and *n* − 1 = 29 degrees of freedom; $\bar{N}$ is the arithmetic mean, calculated from the following equation:

(2) $$\bar{N}=\frac{1}{n}\sum_{i=1}^{30}N_{i};$$

and *S_n_* is the standard deviation:

(3) $$S_{n}=\sqrt{\frac{1}{n-1}\sum_{i=1}^{30}{\left(N_{i}-\bar{N}\right)}^{2}}.$$

The dispersion of the results, expressed by $\pm \frac{t}{\sqrt{n}}S_{n}$, was ±0.3 ÷ 0.6 μm. The obtained arithmetic mean values are collected in Table 1, labeled according to the applied parameters as follows:

- The first character corresponds to the respective coordinate (X, Y, or Z).
- The second character denotes the respective probe tree (1, 2, or 3), as specified in Figure 2.
- The third character indicates the direction of the probe: *x*—along the *x*-axis of the PCS (along the shaft axis), with *B* = 180°; *y*—along the *y*-axis of the PCS, perpendicular to the shaft axis, with *B* = 90°.

From Table 1, it can be noted that during measurements with the vertical probe position at the recommended angle *A* = 0°, the angle *B* also had an effect on the results. When the angle was *B* = 180° (the third letter in the code of the experiment was *x*), the results for the X- and Z-axes were always greater than those marked with *y* (*B* = 90°). On the contrary, the results for the Y-axis at *B* = 180° (marked with *x*) appeared to always be smaller than the respective results obtained at *B* = 90° (marked with *y*). Notably, the Z coordinates exhibited the smallest differences of 0.1 μm.

However, the effect of angle *B* became more significant when the probe was declined from the vertical position, including the case of the Z-axis. When the declination angle was *A* = 15°, the *B*-dependent differences in the values of X and Z increased up to almost 1 μm, and up to almost 2 μm for the Y values. The results for Z appeared to be positive for *B* = 90° and negative for *B* = 180°.

It seems that the negative results for the Z values in the experiments Z1y, Z2y, and Z3y could be attributed to the sliding movement of the probe ball tip on the surface of the measured shaft when the stylus was positioned perpendicularly to its axis. On the other hand, positive deviation from the expected value (Z1x, Z2x, and Z3x) appeared when the stylus was positioned along the *x*-axis. This can be explained by considering that the ideal contact point of the probe ball tip with the shaft surface, identified for a force of 0 N, would move upward when the contact force reached its real value due to the deflection of the stylus.

**Figure 5:**
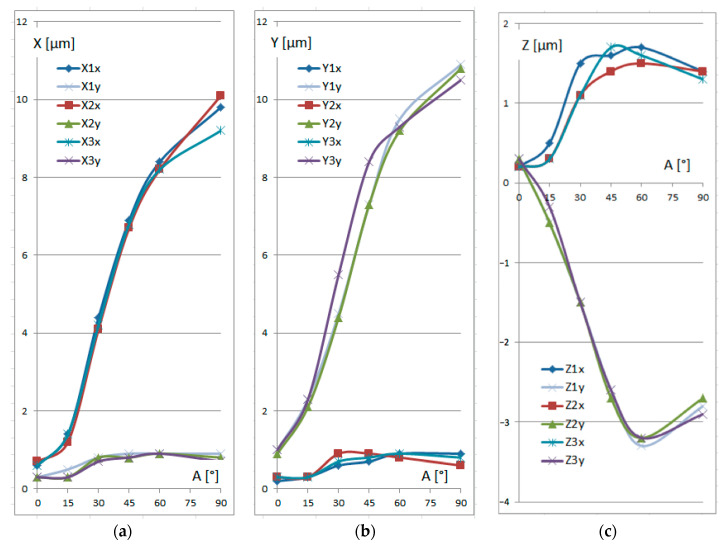
Results of identification of the (0,0,0) point for different probe trees and rotation angles *A* for the respective coordinates: (**a**) X coordinate; (**b**) Y coordinate; (**c**) Z coordinate.

Compared to the maximum permissible error, *MPE_E_*_0_, these differences appeared to be acceptable, since they had no significant effect on the overall accuracy of the measurements. Thus, if the operator follows the respective recommendations and the probe declination is no more than a few degrees, point identification can be performed with satisfactory accuracy. However, further increases in angle *A* caused dramatic increases in the differences between the expected true values of the coordinates and the actual measurement results. This is clearly seen in the graphical presentation of the results in Figure 5.

It is worth noting that Figure 5a,b contain the results of different orientations, which are similar but mirrored. Thus, the error in the OX direction measured along the OY direction is the same as the error in the OY direction measured along the OX direction. At larger values of angle *A*, the effect of angle *B* became much more significant. In the case of the X coordinate, angle *B* = 180° generated much larger differences than angle *B* = 90°. When identifying the Y coordinate, angle *B* = 90° caused large differences compared to the results at angle *B* = 180°. A similar effect can be seen for the Z coordinate, but to a smaller extent. Interestingly, the three different probe trees exhibited no significant differences in measurement results.

Thus, under the conditions specified in Section 2, measurement of the cylindrical surface was accompanied by significant errors when the stylus was positioned horizontally along the shaft axis (*A* = 90°, *B* = 180°). In this situation, the circumferential points of the probe ball tip were involved in the measurement process, instead of the recommended point laying on the axis of the stylus.

**Figure 6:**
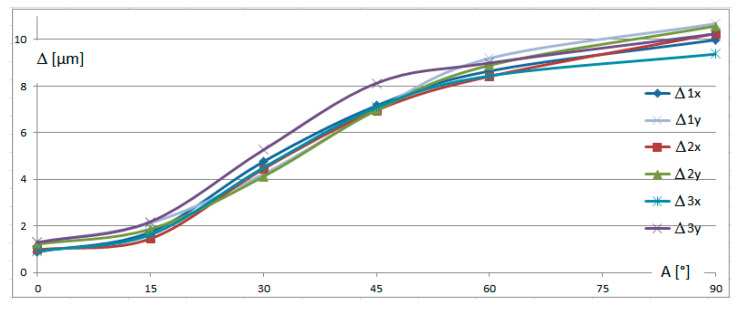
Diagram of relative errors Δ depending on the rotation angle *A*. Explanation of the respective notions of the lines is given in Table 2.

For the presented results, identification errors Δ were calculated, representing the mean square of the differences between the expected coordinates (0,0,0) and the actual measured X, Y, and Z values. These are graphically presented in Figure 6, while Table 2 contains the explanation of the graph contents.

The relative error was smallest for small declinations, when angle *A* was close to the recommended value of *A* = 0°. For larger angles *A*, the error Δ became larger and more differentiated, depending on angle *B* and the swivel length. Thus, it can be concluded that for the recommended vertical positioning of the stylus, the relative error generated by the swivel length and stylus direction can be omitted. However, when the measurement conditions necessitate the use of significant declination of the stylus above 10–15°, some additional errors may appear.

It should be emphasized that the negligible effect of the swivel length appeared to be repeatable for the two probes of similar lengths, *L_S_*_2_ = 257.35 mm and *L_S_*_3_ = 253.85 mm, as well as for the significantly different one, *L_S_*_1_ = 173.35 mm. Nevertheless, in order to enhance the comprehensiveness and reliability of the study, it is planned to verify the effect of swivel length using other probes in the subsequent research programs.

In industrial coordinate measurements in CNC mode, operators often deal with atypical situations where it is impossible to keep the optimal or recommended measurement conditions. This may occur in cases of specific, complex automotive and aircraft components, but also in shaft-type parts when measuring hard-to-reach grooves, grinding undercuts, splines, conical surfaces, etc. Similarly, the problem may appear when no calibrated probe with an appropriate angle of rotation is available at the moment.

In particular, when the probe was aligned along the measurement axis (*A* = 90°), which was acceptable but not recommended, the deviation was unexpectedly large—almost five times the maximum permissible error, *MPE_E_*_0_. Presumably, it could happen due to the unpredictably disadvantageous superposition of the calibration and measurement parameters. Among other factors, the allowable probe deflection and CNC movement speed, which were different from the recommended ones, might have had a negative effect, which needs to be investigated in future research. In addition, at larger angles, this effect could be multiplied by the highly possible sliding movement of the probe tip from the correct contact point. Thus, high-precision measurements require careful checking of the parameters and possible errors when the recommended values cannot be maintained.

The above analysis proved that slight deviations from the recommendations on typical measurement conditions are acceptable and will have a negligible effect on the measurement accuracy, irrespective of the swivel length. This finding is important because in industrial practice, the complexity of a measured component may make it impossible to follow the recommendations strictly. It is particularly important in the context of the I4.0 concept, where CMMs play a key role in inspection planning systems [38] and in the implementation of customized quality inspection cycles [39]. The required accuracy and reliability of CMM measurements can be ensured despite the decline in the stylus by a few degrees, without the need for additional individual analysis.

## 4. Additional Experiments

**Figure 7:**
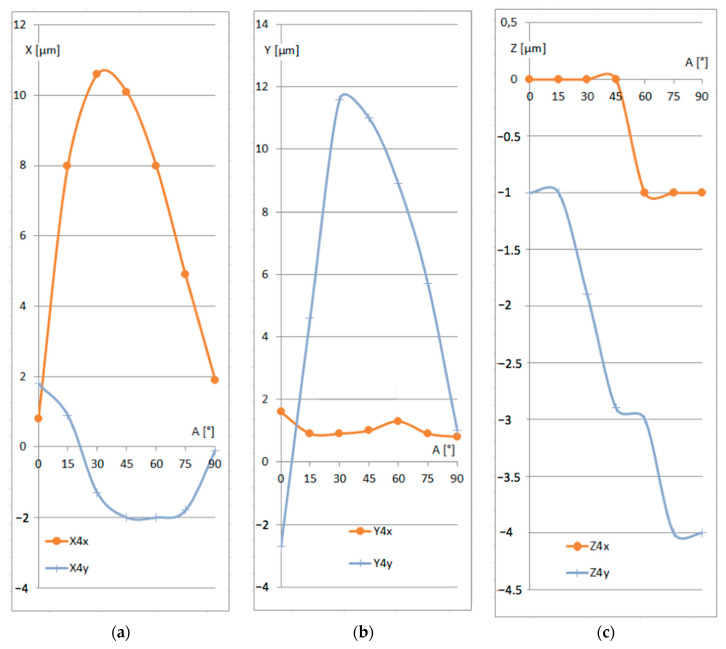
Additional results of the identification of the (0,0,0) point for different rotation angles *A* for the respective coordinates: (**a**) X coordinate; (**b**) Y coordinate; (**c**) Z coordinate.

**Figure 8:**
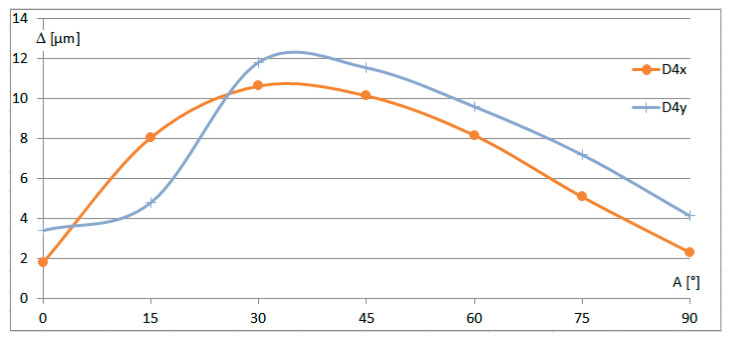
Diagram of relative errors Δ for the additional experiments, depending on the rotation angle *A*.

In response to the questions raised by the Reviewers, a series of additional measurements were performed using a different type of CMM. To avoid any conflicts of interest, we do not indicate the CMM model. However, the application of a different CMM under exactly the same conditions appeared to be impossible. First of all, the operational system did not allow for keeping the same settings as in the experiments described above. Moreover, the operator was not willing to change the calibration parameters for the experiments because it would interrupt the normal work of the CMM in the industrial conditions. Thus, it was only possible to imitate the measurement strategy and to use the same shaft, even though the exact identification of the same point was obviously impossible. The respective diagrams are shown in Figure 7 and Figure 8.

Given the different parameters of the repetitions, it would be expected to obtain different trends than those shown in Figure 5 and Figure 6. Especially large differences are seen in the ranges of larger values of angle *A*. In the experiments described in Section 3, the trend lines for X and Y slightly changed their declinations at *A =* 45°, which corresponded with the extreme of the Z trend. In the additional experiments illustrated in Figure 7 and Figure 8, extremes are seen in the X and Y diagrams, while the Z trend exhibits a change in declination. One might have expected this sort of trend to appear in the first experiments as well, but at the present stage, it is impossible to extract the main factors influencing the differences in the results. In fact, the unpredictable superposition of those uncontrolled factors likely resulted in such a large differences.

Notably, the diagram of relative errors Δ for the additional experiments shown in Figure 8 exhibits extremes at angles of 30–45°. The smallest errors occurred at the recommended value of *A =* 0°, as well as at the acceptable value of *A =* 90°, defined for high-precision measurements.

## 5. Conclusions

The research results indicate that in some metrological cases, when an operator is forced to perform tactile CMM measurements with head positions different from the recommended ones, a significant decrease in accuracy may take place. Under the chosen experimental conditions described in this paper, the coordinates of the points could be identified with negligible additional error only when the angle *A* differed from the recommended angle of 0° by a few degrees. This sort of situation may appear at the initial stage of the CNC measurement process, when the measured surface cannot be easily reached, or when replacement of the stylus would take too much time and a quick operation is required. The uncertainty of the obtained results will most likely be placed in the range declared by the CMM manufacturer or by the calibration certificate.

However, the experiments demonstrated that larger probe declination angles introduced large deviations from the expected values of the point coordinates. Specifically, when the stylus was positioned along the axis of the measured shaft, i.e., *A* = 90°, the deviations from the true values reached up to 10 μm—almost five times larger than the maximum permissible error, *MPE_E_*_0_. On the other hand, for the recommended vertical positioning of the stylus, the relative error generated by the swivel length and stylus direction was found to be negligible.

It is very important to emphasize that the present case study revealed large differences in the error trends in additional experiments with another CMM. These differences might have emerged from the different constructions, software, and other uncontrolled factors and their superposition. Hence, it is likely that the effects of probe angle and swivel length on contact point identification should be assessed individually. For any sort of generalization, a wide range of CMMs should be tested. In particular, it is planned to perform systematic testing of a larger number of different swivel lengths.

The significance of the findings should not be underestimated, since it is quite common in industrial practice to collect the probing point on the shaft with the stylus positioned this way. As a result, the fitting element can differ significantly from the true one, and the coordinates of the circle’s center calculated from it may be erroneous. Considering the high geometrical accuracy requirements in the automotive and aerospace industries, especially in context of the I4.0 concept, the introduction of such a significant additional measurement error is absolutely unacceptable.

## Figures and Tables

**Table 1:** Arithmetic mean values of the X, Y, and Z [μm] coordinates of the point (0,0,0) for different rotation angles *A* of the probes from three probe trees and different positions related to the shaft axis.

Experiment Code	*A* = 0°	*A* = 15°	*A* = 30°	*A* = 45°	*A* = 60°	*A* = 90°
X1x	0.6	1.4	4.4	6.9	8.4	9.8
X1y	0.3	0.5	0.8	0.9	0.9	0.9
X2x	0.7	1.2	4.1	6.7	8.2	10.1
X2y	0.3	0.3	0.8	0.8	0.9	0.8
X3x	0.6	1.4	4.2	6.8	8.2	9.2
X3y	0.3	0.3	0.7	0.8	0.9	0.7
Y1x	0.2	0.3	0.6	0.7	0.9	0.9
Y1y	1.0	2.3	4.5	7.3	9.5	10.9
Y2x	0.3	0.3	0.9	0.9	0.8	0.6
Y2y	0.9	2.1	4.4	7.3	9.2	10.8
Y3x	0.3	0.3	0.7	0.8	0.9	0.8
Y3y	1.0	2.3	5.5	8.4	9.3	10.5
Z1x	0.2	0.5	1.5	1.6	1.7	1.4
Z1y	0.3	−0.5	−1.5	−2.6	−3.3	−2.8
Z2x	0.2	0.3	1.1	1.4	1.5	1.4
Z2y	0.3	−0.5	−1.5	−2.7	−3.2	−2.7
Z3x	0.2	0.3	1.1	1.7	1.6	1.3
Z3y	0.3	−0.3	−1.5	−2.6	−3.2	−2.9

**Table 2:** The positions of the probe and the probe tree numbers, corresponding with the results presented in Figure 6.

Line in Figure 6	Probe Tree (See Figure 2)	Angle *B* (See Figure 1)
∆1x	1	*B* = 180°
∆1y	1	*B* = 90°
∆2x	2	*B* = 180°
∆2y	2	*B* = 90°
∆3x	3	*B* = 180°
∆3y	3	*B* = 90°

## Data Availability

The original contributions presented in this study are included in the article. Further inquiries can be directed to the corresponding author.
